# Total IgE in tears accurately reflects the severity and predicts the prognosis of seasonal allergic conjunctivitis

**DOI:** 10.1002/clt2.12139

**Published:** 2022-03-25

**Authors:** Jiayu Bao, Lei Tian, Yifan Meng, Binge Wu, Jingyi Wang, Jing He, Qiyan Shao, Chengshuo Wang, Ying Jie, Luo Zhang

**Affiliations:** ^1^ Beijing Institute of Ophthalmology Beijing TongRen Eye Center Beijing Key Laboratory of Ophthalmology and Visual Sciences Beijing Tongren Hospital Capital Medical University Beijing China; ^2^ Department of Otolaryngology Head and Neck Surgery Beijing TongRen Hospital Capital Medical University Beijing China; ^3^ Department of Ophthalmology the Second Affiliated Hospital of Baotou Medical College Inner Mongolia University of Science and Technology Beijing China; ^4^ Beijing Key Laboratory of Nasal Diseases Beijing Institute of Otolaryngology Beijing China; ^5^ Department of Allergy Beijing TongRen Hospital Capital Medical University Beijing China

**Keywords:** diagnosis, prognosis, seasonal allergic conjunctivitis, tears, total IgE

## Abstract

**Background:**

Although immunoglobulin E (IgE) increases significantly in tears and serum during seasonal allergic conjunctivitis (SAC), it is unclear whether tear total IgE can reflect the severity and prognosis of SAC more accurately than serum total IgE. We aimed to investigate the usefulness of measuring the total IgE in tears to evaluate the severity and determine the treatment of SAC.

**Methods:**

This prospective, nonrandomized study involved 55 patients with SAC and 10 age‐ and sex‐matched healthy controls. Serum and tears were collected before and after treatment to analyze the total IgE. SAC patients received the same topical anti‐allergy treatment and were followed‐up every 2 weeks for 1 month. The relationship of tear and serum total IgE concentrations with pollen concentrations and symptom severity before and after treatment was assessed.

**Results:**

The total IgE concentration in tears was higher in SAC patients than in healthy participants with significant correlations between tear and serum total IgE concentrations. The total IgE concentration in tears, but not in serum, correlated with the pollen concentration and severity of ocular symptoms and reactions in SAC. Treatment‐associated improvements in symptoms and reactions in SAC correlated with decreased concentrations of the tear total IgE. Patients with disease recurrence following treatment demonstrated significantly higher tear total IgE concentrations than patients with no recurrence.

**Conclusion:**

The total tear IgE level can indicate the severity and predict the prognosis of SAC more accurately than the serum total IgE.

## 1 INTRODUCTION

Allergic conjunctivitis (AC), an inflammatory response of the conjunctiva to allergens such as pollen, animal dander, and other environmental antigens, affects 15%–40% of the population.^1^, ^2^, ^3^ Seasonal allergic conjunctivitis (SAC) is the most common form of ocular allergy and accounts for 90% of AC cases.^4^, ^5^ SAC has a considerable impact on patients' quality of life and comfort, and results in a substantial burden on the economy through health care costs and the reduction in productivity.^6^ Signs and symptoms of SAC include redness, periocular itching, epiphora, burning, stinging, photophobia, watery discharge, and swollen or dry eyes, with differing degrees of severity and variable duration.^7^, ^8^

Since its discovery more than 50 years ago, immunoglobulin E (IgE) has been shown to be involved in a wide variety of immunological diseases, including allergic disease.^9^, ^10^, ^11^ Despite being the least abundant immunoglobulin in normal human serum, IgE plays a key role in SAC.^12^, ^13^ IgE, when secreted in tears, binds to fragment crystallizable (Fc) receptors on mast cells.^14^ In the case of re‐exposure to allergens, it leads to mast cell degranulation release of mediators such as histamine, resulting in chemosis and ocular itching.

The diagnosis of ocular allergies is primarily clinical, although laboratory tests can be useful in supporting the diagnosis.^6^ At present, laboratory tests for AC mainly include cytological examination, skin prick tests, and total IgE antibody detection.^15^, ^16^, ^17^ The detection of eosinophils is a complex procedure with a low detection rate of approximately 60% in conjunctival scrapings from patients with mild allergic conditions.^18^ Skin prick tests are invasive and require multiple pricks with small volumes of allergens.^8^ Owing to the large ocular surface reaction caused by the conjunctival provocation test, the clinical application of this test is limited.^19^, ^20^ Moreover, although serum total IgE tests are recommended for etiological diagnosis, the test does not provide information on the pathogenesis and severity of the disease.^21^ Given its high prevalence, it is therefore important to measure the total IgE in tears to help diagnose SAC.^22^ However, assessing the severity of SAC by measuring the total IgE in tears remains to be elucidated. We hypothesized that determining the total IgE levels in tears in patients with SAC, relative to the serum total IgE, may allow for better evaluation of the severity of SAC and allow for prediction of the prognosis of the disease. Thus, the aim of this study was to explore whether the total IgE levels in tears or serum can better reflect the severity of the disease in providing a baseline for the diagnosis and treatment of SAC.

## 2 MATERIALS AND METHODS

### 2.1 Study design

This was a prospective, non‐randomized study conducted at the Second Affiliated Hospital of Baotou Medical College, Inner Mongolia University of Science and Technology. The study was approved by the Institutional Review Committee of the hospital and carried out in accordance with the Helsinki Declaration of 1975 and the 1983 amendments (ethics number: HX‐015). Each individual provided written informed consent before participating in the study.

### 2.2 Participants

**FIGURE 1:**
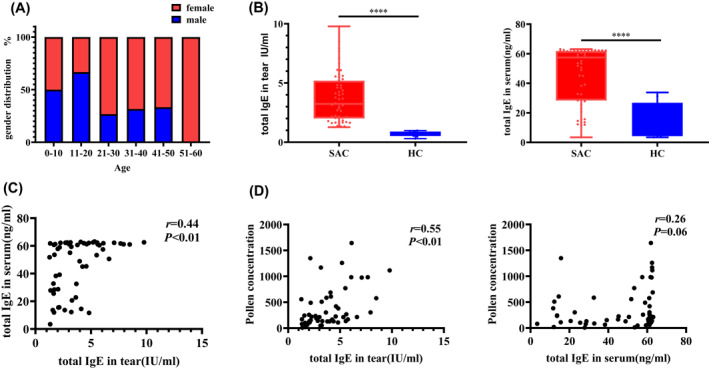
(A) Age distribution of the enrolled patients. (B) Comparison of the baseline levels of the total IgE concentration in tears (left panel) and serum (right panel). The differences between groups were compared using the two‐tailed Mann–Whitney *U* test. (C) Correlation between tear and serum total IgE levels. (D) Correlation between pollen concentration and total IgE in tears (left panel), and total IgE in serum (right panel). HC, healthy control group (*n* = 10), AC: SAC group (*n* = 55). The association between variables was studied by calculating the Spearman's correlation coefficient

Fifty‐five patients (19 men and 36 women; mean age: 31 years; age range: 7–56 years (Figure 1A)) with SAC were enrolled in the study from March 2020 to August 2020 (Table 1). All participants had a positive serum total IgE test result. A clinical diagnosis was made according to subjective symptoms (including ocular itching, redness, tearing, foreign body sensation, and burning sensation) and objective symptoms (including palpebral conjunctiva hyperemia, palpebral conjunctiva swelling, bulbar conjunctiva hyperemia, bulbar conjunctiva chemosis, palpebral conjunctiva follicle, and palpebral conjunctiva follicles). None of the participants had received local or systemic medication for 6 weeks prior to the study. Patients were excluded if they had dry eye, atopic eyelid conjunctivitis, atopic keratoconjunctivitis, or vernal keratoconjunctivitis. Similarly, patients who used contact lenses, and those with a history of cataract surgery, corneal refractive surgery, or infectious conjunctivitis were also excluded, as were patients with other allergic diseases, including allergic rhinitis, allergic asthma, and allergic dermatitis.^23^, ^24^ Patients over 60 years and under 5 years old were also excluded. Participants with chronic diseases, mental disorders, on medication, or who abused drugs were excluded. Furthermore, participants on continuous drugs for a chronic disease or smoking as well as those who had parasitic infections were also excluded. In addition, 10 healthy age‐ and sex‐matched participants with no history of allergic diseases were recruited as the healthy control group.

**TABLE 1:** Demographic characteristics of the patients

Characteristic	*n* = 55
Age (years), mean ± SEM	31.16 ± 1.56
Sex, female/male, No (%)	36 (65.45)/9 (34.55)
Allergen sensitization	Pollen
Duration of SAC (year), mean ± SEM	9.02 ± 0.55
Smoking history, *N*	0
Drinking history, *N*	0
Family history, *N*	15
Pet ownership, *N*	35
BMI (kg/m^2^), mean ± SEM	21.87 ± 0.29
Place of birth	
City	35
Country	20
Premature infant	3
Total serum IgE (ng/ml), mean ± SEM	45.99 ± 2.60
Total tear IgE (IU/ml), mean ± SEM	3.74 ± 0.27

Abbreviation: *N*, number.

### 2.3 Measurement of the pollen concentration

Pollen concentrations in areas of patients' residence were recorded using the Durham pollen collector,^25^ which was placed 14–16 m above from the ground. The sampling slides were replaced every 24 h, and the collected samples were stained with alkaline fuchsine before microscopic analysis. The unit for pollen concentration was grains per thousand square millimeters.

### 2.4 Diagnosis of SAC

The diagnosis of AC was based on the results of a slit‐lamp examination and an allergen test. The diagnosis was made by an ophthalmologist based on published guidelines, and successful treatment of conjunctivitis was noted when a patient reported the symptom of ocular itching to have disappeared or to occur only occasionally (score = 1) which was judged to be significantly improved.

### 2.5 Symptom and sign scores of allergic conjunctivitis

All participants completed a questionnaire at the first visit and at each follow‐up visit, and their symptoms were scored with a doctor's assistance. The symptom scores were obtained for both eyes for each patient; however, for the purpose of this study, only the score from the right eye was used for analysis. The doctor who assisted patients with their symptom score also scored the ophthalmic results of each patient's slit‐lamp examination (Supplemental Table S1).^26^, ^27^, ^28^, ^29^, ^30^

### 2.6 Measurement of total IgE in tears

Three microliters of tears were collected from each patient using a capillary pipette at the first visit and at each of the follow‐up visits. The lateral flow immunoassay was utilized to quantitatively determine the amount of human IgE in the tears. Nonstimulated tear fluids from the lateral canthus of one eye were collected using a disposable microcapillary fluid collector (3 μL; Seninda Biomedical Corporation, China); the tear fluids were stored in a 0.5 ml centrifuge tube, and the sample was frozen immediately at −80°C. A total of 2.2 μL of the sample was dispensed into the test card (Seninda Biomedical Corporation, China), and three full drops of buffer were added. The test card was allowed to rest undisturbed for 10 min, and then the i‐ImmunDx Analyzer was used for reading. The test was performed separately for each eye.

### 2.7 Determination of the serum total IgE

A total of 2 ml of blood was collected from each patient at the first visit. The serum was separated and stored by freezing at −80°C until it was analyzed. The serum total IgE concentration in the serum sample was detected using an ELISA kit (BMS2097, Thermo Fisher, USA) and quantified according to the manufacturer's instructions.

### 2.8 Treatment and follow‐up

All patients received the same topical anti‐allergy treatment. The drugs used were fluorometholone eye drops, olopatadine eye drops, and sodium hyaluronate eye drops. All drugs were used according to the manufacturer's instructions. After the first visit, the participants were instructed to return to the consulting room for two further visits (one visit every 2 weeks). Thereafter, they were followed‐up via three telephone consultations every other month. Recurrence was defined as recurrence of the disease during the follow‐up period.

### 2.9 Statistical analysis

GraphPad 8.0 software and SPSS 18.0 software were used for analysis and mapping. The differences between groups were compared using the two‐tailed Mann–Whitney *U* test. Any associations between variables were calculated using Spearman's correlation coefficient. The level of significance was set at *p* < 0.05. Data are presented as the mean ± standard error of the mean (SEM). *p < 0.05, **p < 0.01, ***p < 0.001, ****p < 0.0001.

## 3 RESULTS

### 3.1 Levels of IgE in tears and serum increased in SAC

Fifty‐three of the 55 participants with SAC attended the two follow‐ups, during which photographs of the surface of the eyes were taken for further assessment. The baseline concentrations of total IgE in tears and in the serum of patients with SAC were significantly higher than those of control participants (*p* < 0.01; Figure 1B). Spearman's correlation analysis showed that serum total IgE positively correlated with tear total IgE (*r* = 0.44, *p* < 0.01; Figure 1C). Furthermore, there was a significant correlation between pollen concentration and the total IgE in tears (*r* = 0.55, *p* < 0.01) but not between pollen concentration and serum total IgE (*r* = 0.26, *p* = 0.06; Figure 1D).

### 3.2 Total IgE in tears positively correlated with the severity of symptoms

**FIGURE 2:**
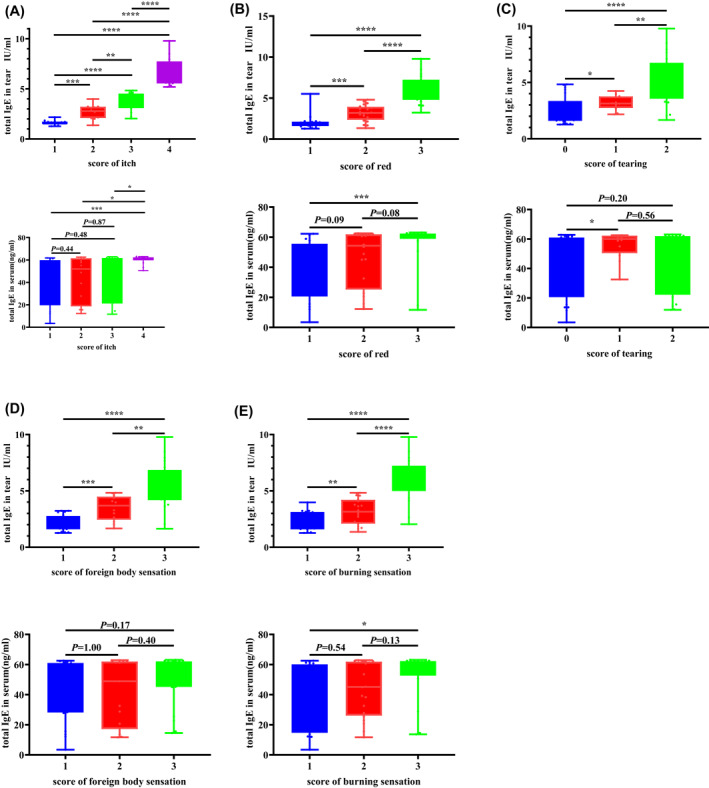
Analysis of the correlation between the tear total IgE or serum total IgE concentration and different symptom scores. The symptoms analyzed included ocular itching (A), redness (B), tearing (C), burning sensation (D), and foreign body sensation (E). The differences between groups were compared using the two‐tailed Mann–Whitney *U* test

Assessment of the correlation between the total IgE or serum total IgE concentration and the ocular itch score revealed that the total IgE concentration in tears, but not the serum total IgE concentration, was significantly positively correlated with the itch score (Figure 2A). Similar results were also noted for correlations between the total IgE or serum total IgE concentration and tearing score, redness score, foreign body sensation score, and burning sensation score (Figure 2B–E).

### 3.3 IgE in tears positively correlated with the severity of signs

**FIGURE 3:**
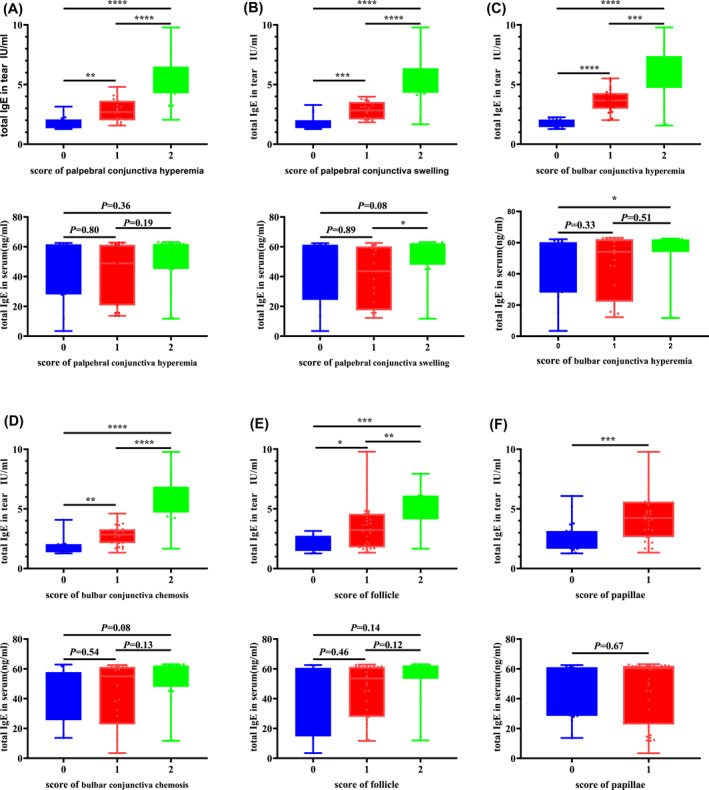
Analysis of the correlation between the tear total IgE or serum total IgE concentration and scores for different conjunctival reactions in patients with SAC. We analyzed conjunctival reactions including palpebral conjunctiva hyperemia (A), palpebral conjunctiva swelling (B), bulbar conjunctiva hyperemia (C), bulbar conjunctival chemosis (D), palpebral conjunctiva follicle (E), and palpebral conjunctiva papillae (F). The differences between groups were compared using the two‐tailed Mann–Whitney *U* test

The total IgE concentration in tears, but not the serum total IgE concentration, was also found to be significantly increased with an increase in palpebral conjunctiva hyperemia score (Figure 3A). While significant correlations were noted between total IgE concentrations in tears and the palpebral conjunctiva swelling score, bulbar conjunctiva hyperemia score, bulbar conjunctiva chemosis score, palpebral conjunctiva follicle score, and palpebral conjunctiva papillae score, the serum total IgE concentration showed a significant difference only in patients with severe disease and in patients without the apparent conjunctival reaction (Figure 3B–F).

### 3.4 Tear IgE correlated with the speed of improvement and recurrence of SAC

**FIGURE 4:**
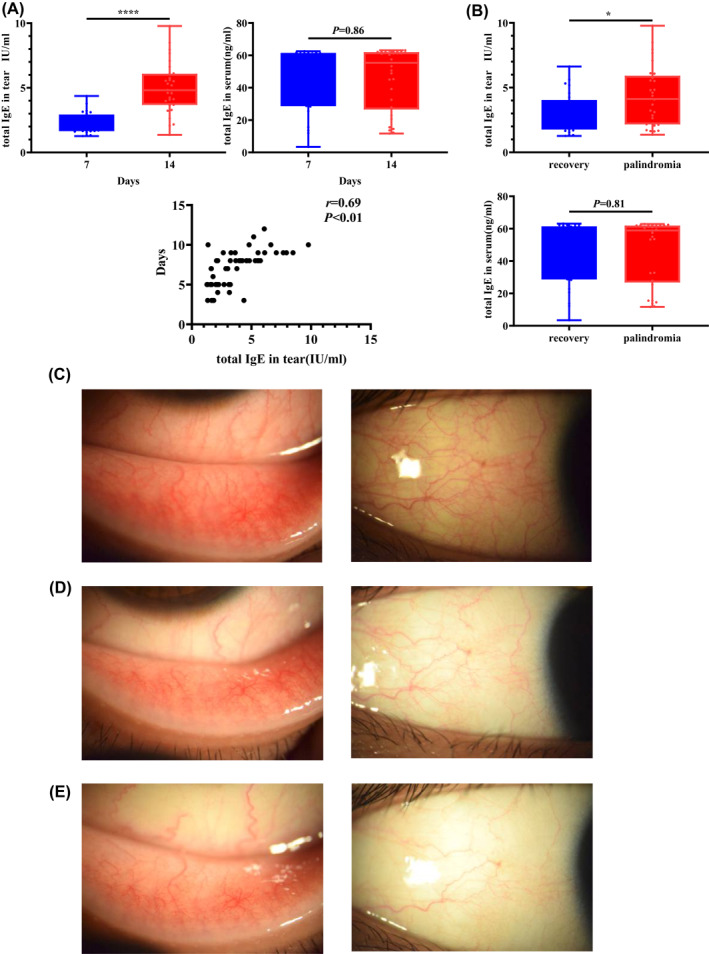
(A). Patients who improved within 7 days and those who improved after 7 days following treatment were categorized into two separate groups. The Mann–Whitney *U* test was used to analyze the difference in the total IgE concentration between the two groups. Spearman's correlation analysis was used to analyze the correlation between the total IgE concentration measured at the first visit and the number of days needed for improvement. (B) After the follow‐up, the subjects were divided into a recurrence group (palindromia) and a non‐recurrence group (recovery). The Mann–Whitney *U* test was used to analyze the difference between the total IgE concentrations of both groups. Typical findings for a 37‐year‐old male SAC patient with (C) lower palpebral conjunctival and bulbar conjunctival reactions at the first visit; moderate hyperemia and edema are present. The concentration of total IgE in the tears was 7.94 IU/ml. (D) Lower palpebral conjunctival and bulbar conjunctival reaction at the first follow‐up visit; mild hyperemia and edema are present. The total IgE concentration was 2.10 IU/ml. (E) Lower palpebral conjunctival and bulbar conjunctival reactions at the second follow‐up visit. The total IgE concentration was 0.52 IU/ml

Patients were separated into two groups, those who improved within 7 days and those who improved after 7 days. The total IgE in the tears of patients who improved within treatment for 7 days was significantly lower than that in the tears of the patients who improved after treatment for 7 days (*p* < 0.01; Figure 4A). In contrast, there was no significant difference in the serum total IgE concentration between the two groups. Spearman's correlation analysis further demonstrated that the total IgE concentration in tears before treatment positively correlated with the number of days until symptom improvement (*p* = 0.86; Figure 4A). Assessment of the total IgE in tears and serum of patients who had recovered or relapsed following treatment showed that total IgE concentrations in tears at the initial visit were significantly higher in patients who relapsed after drug discontinuation than in patients who had recovered (*p* = 0.03), whereas serum total IgE concentrations were not significantly different between the two groups (*p* = 0.81; Figure 4B). A typical case is shown in Figure 4.

### 3.5 The change in tear IgE can reflect the evolution of disease severity

**FIGURE 5:**
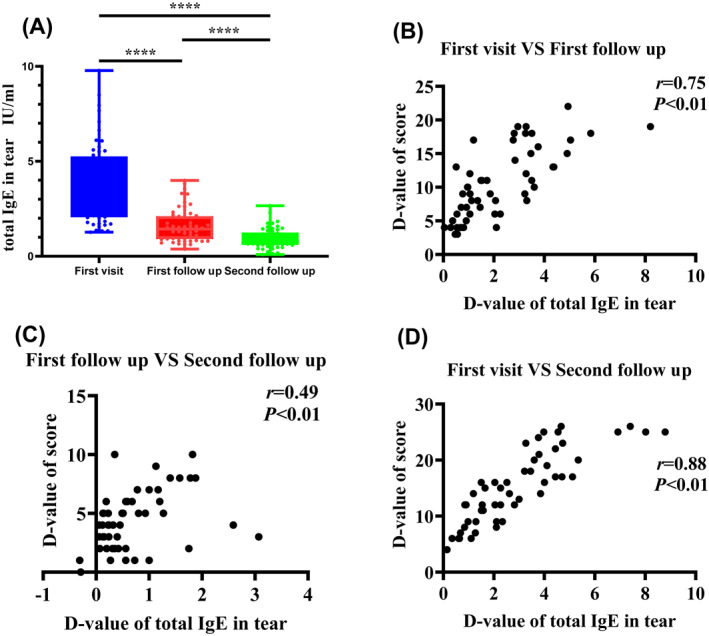
(A) Difference in the tear total IgE concentration compared at each follow‐up visit. (B–D) Total clinical scores, obtained by adding the scores of each symptom and conjunctival reaction. The difference in the total IgE concentration in tears was obtained by subtracting the total IgE concentration measured at each visit. The correlation between the difference in the total IgE concentration and the difference in the total clinical score was analyzed using Spearman's correlation analysis. The D‐value of IgE is the difference between the total IgE concentrations in tears measured at the two visits. The D‐value of the score is the difference between the total clinical scores recorded at the two visits

In this study, 53 participants completed two follow‐up visits. The results showed that with patient improvement, the concentration of total IgE in tears decreased significantly (*p* < 0.01; Figure 5A). The correlation analysis further showed that the difference between the total IgE concentrations in tears measured at the first visit and the first follow‐up visit positively correlated with the difference between the total clinical scores recorded at the two visits (Figure 5B). Similarly, there was a significantly positive correlation between the change in the tear total IgE concentration and the difference in clinical score at the every 2‐week follow‐up (Figure 5C,D).

## 4 DISCUSSION

At present, the diagnosis of SAC is based on the evaluation of symptoms and ocular reactions of the patient as well as laboratory examination. However, the current laboratory examination methods are inadequate and cannot be used to evaluate the severity of SAC. Therefore, we sought to conduct an in‐depth study of tear IgE to help find a detection method that can reflect the severity and prognosis of SAC. In the present study, we analyzed the correlation between total IgE and disease severity and prognosis and found that compared with the serum total IgE concentration, the tear total IgE concentration could better reflect the severity of SAC and development of the disease.

In our study, the tear total IgE levels in patients with SAC were substantially higher than those in healthy participants, suggesting that the tear total IgE levels reflect the immune response of patients similar to the serum total IgE levels. In patients with SAC, allergens can enter the eyes via the conjunctiva and produce local IgE, which infiltrates the tears through the blood tear barrier, resulting in increased IgE levels in tears.^31^ This study additionally demonstrated that the concentration of serum total IgE positively correlated with the concentration of tear total IgE. However, the correlation coefficient for tear and serum total IgE was relatively low (*r* = 0.44). It is possible that this is because SAC is an allergic disease, which is dominated by local immunity with more pronounced local symptoms, whereas serum total IgE levels usually reflect the state of the immune response of the whole body. However, other studies^32^, ^33^ that investigated the association between the total IgE in tears and serum have found conflicting data. While a study by Kari et al^32^ was unable to demonstrate a correlation between serum total IgE and tear total IgE levels in patients with SAC, a study by Mimura et al.^33^ which investigated patients with moderate to severe SAC, found a significantly high correlation (*r* = 0.71) between the total IgE in tears and serum. However, as SAC is a disease with predominantly ocular symptoms, testing for the tear total IgE is more relevant, and future studies on SAC should concentrate on the localized immune response.

The current study also investigated any associations between pollen concentration and total IgE in tears and serum. Interestingly, the tear total IgE positively correlated with the pollen concentration, indicating that higher pollen concentrations are likely to lead to the presence of higher total IgE concentrations in tears. Furthermore, there was no correlation between the pollen concentration and serum total IgE, indicating that allergens stimulate the conjunctiva to produce local total IgE rather than lead to increased serum total IgE. Indeed, there is evidence showing that stimulation of the conjunctiva by pollen results in various inflammatory cells infiltrating the conjunctiva and releasing different cytokines that regulate the synthesis of total IgE.^34^ Among these cytokines, interleukin 4 (IL‐4) promotes the synthesis of total IgE, whereas interferon‐gamma (IFN‐γ) inhibits IL‐4‐induced total IgE.^35^, ^36^ However, the correlation coefficient for tear total IgE and pollen concentrations was relatively low (*r* = 0.55). It is possible that this is because we analyzed the total IgE in tears and not specifically pollen IgE. Recently, Yamana et al.^37^ also found that pollen‐induced AC can be caused by pollen sensitization in the conjunctiva since pollen‐specific IgE antibodies have a higher total tear IgE positive rate than other antigens. This further confirms our results. Nonetheless, we will focus more on tear‐specific IgE in future studies.

This study demonstrated that tear total IgE was also able to better reflect the severity of SAC than serum total IgE, particularly as an increase in symptom scores and reactions, including itching, tearing, and edema coinciding with significantly increased concentrations of tear total IgE. This suggests that the tear total IgE is a key factor in aggravating ocular symptoms. When mast cells in close proximity to the itch‐sensory nerve fibers are activated by IgE and have undergone degranulation, a variety of itch‐inducing molecules or pruritogens, such as histamine, are released and bound to their respective receptors on terminal axons. This leads to signals that pass through the central nervous system to the brain, where these signals are perceived as an itch.^35^, ^38^, ^39^, ^40^ Thus, a higher concentration of tear total IgE leads to a stronger itch sensation. Additionally, activation of mast cells by IgE leads to induction of vasodilation, promotion of vascular permeability, recruitment of inflammatory cells, facilitation of an adaptive immune response, modulation of angiogenesis, and fibrosis.^41^, ^42^, ^43^ Thus, the tear total IgE plays a key role in SAC, as a higher concentration may lead to more pronounced eye symptoms. In comparison, the serum total IgE concentration in the present study only showed a significant difference only in patients with severe disease and patients without apparent signs of SAC. Furthermore, as an increase in blepharo conjunctival vascular permeability may contribute to the local release of IgE into tears, it is likely that tear total IgE levels are more capable of reflecting the local immune state of allergies than serum total IgE. Recently, Neil et al.^44^ consolidated the measured/exuded tear IgE ratio with the eosinophil cationic protein level, Th1/Th2 cytokines, and main tear proteins to better understand tear inflammatory mediator. They found that the measurement of the IgE ratio in tears is a useful and discriminant tool to confirm the diagnosis of ocular allergy, particularly in chronic or persistent forms when the etiology is difficult to determine based on clinical criteria and allergic anamnesis. Their findings are similar to our results. However, the researchers did not analyze the clinical symptoms; thus, future studies should investigate the IgE ratio in tears and its association with clinical signs and symptoms.

Although SAC is a mostly self‐limiting disease, it often recurs and can persist for many years. In our study, we found that 52.72% (29/55) of patients had recurrent disease and that the total IgE concentration in tears of patients with recurrent illness was significantly higher than that in patients without recurrent disease. Interestingly, a higher tear total IgE concentration at the initial visit was also associated with a longer delay in improvement following treatment. The correlation coefficient for the correlation between the tear total IgE and days until symptom improvement was relatively low (*r* = 0.69). This might be attributed to the small sample size and the fact that each patient has a different level of sensitivity to drug treatment. Moreover, as improvement during the follow‐up period was associated with a significantly decreased concentration of tear total IgE, it is likely that the total IgE concentration in tears may also be useful in determining the prognosis of patients with SAC. Thus, it might be possible to predict the development of the disease by monitoring changes in the tear total IgE concentration. Concurrently, we also noted that the correlation coefficient for the change in tear total IgE concentration and the difference in clinical score at the every 2‐week follow‐up was relatively low (*r* = 0.49). It is possible that with the increase in the follow‐up time, the patient's condition significantly improved. We failed to collect tears immediately after the patient's condition improved, resulting in an incomplete analysis.

The diagnosis of SAC currently includes the evaluation of the symptoms and signs of the patient, as well as laboratory examination. To properly diagnose the disease, the clinician needs to review the history, observe the symptoms and signs, and then supplement these findings with the results of laboratory examination prior to initiating treatment. However, the current laboratory examination methods cannot conveniently obtain the results or be used to evaluate the severity of SAC. Measurement of the tear total IgE levels of patients with SAC could be used as a rapid and immediate diagnostic method. The current study demonstrated the convenience and accuracy of measuring the tear total IgE. Indeed, this measurement has several advantages over measurement of the serum total IgE. In particular, immunochromatography and double antibody Sandwich method are quicker to perform than the standard conventional method and, therefore, may be of considerable benefit to the patient, as SAC may be diagnosed at the patient's first visit. Furthermore, this is a non‐invasive technique that requires only 3 μL of tears to diagnose SAC. Compared with serum, tears also have better stability, are easy to transport and store, and have a specific field application value. Additionally, compared with that of serum total IgE, sensitivity of tear total IgE is higher and can be detected at a low dose and used in micro amounts. Another simple and fast technology called “Allerwatch” can also detect total IgE levels in AC.^45^, ^46^ However, at present, such testing products cannot be purchased in China. “Allerwatch” is a qualitative product, which that can only be used to judge a negative and positive diagnoses of AC. Seinda's i‐immudx IgE test is a quantitative product, that can detect the concentration of IgE in tears. Therefore, it can be used not only for auxiliary diagnosis, but also for evaluating the curative effect.

One of the limitations of this study was that we excluded patients with atopic keratoconjunctivitis because the study focused on SAC. In the future, we will study the relationship between the total tear IgE levels and specific serum IgE levels in patients with atopic keratoconjunctivitis. Further, considering the favorable principle of bioethics, we only collected serum samples of the participants at the first visit to detect the serum total IgE and did not measure its dynamic change. Furthermore, in line with the principle of bioethics, only10 healthy volunteers were included in our study. In future research, we will pay more attention to the consistency of the numbers of the control and experimental groups. Moreover, all included patients had mild to moderate SAC. Therefore, further evaluation is needed for patients with severe AC.

## 5 CONCLUSION

This study demonstrated that the measurement of total IgE levels in tears over serum of patients with SAC could be used as a rapid and immediate diagnostic method, to better evaluate the severity of SAC and predict the prognosis of the disease.

## AUTHOR CONTRIBUTION

**Lei Tian:** Data curation; Methodology. **Yifan Meng:** Data curation; Formal analysis; Methodology. **Binge Wu:** Data curation. **Jingyi Wang:** Data curation; Methodology. **Jiayu Bao:** Data curation; Methodology. **Jing He:** Data curation; Methodology. **Qiyan Shao:** Data curation; Methodology. **Chengshuo Wang:** Conceptualization; Project administration; Writing – review & editing. **Ying Jie:** Funding acquisition; Project administration; Writing – review & editing; **Luo Zhang:** Conceptualization; Methodology; Project administration; Supervision; Writing – review & editing.

## Supporting information

Supplementary Material
