# Supplementary material for: Total IgE in tears accurately reflects the severity and predicts the prognosis of seasonal allergic conjunctivitis
Source: Clin Transl Allergy. 2022 Mar 25;12(3):e12139. doi: 10.1002/clt2.12139 (PMC8967263; doi:10.1002/clt2.12139)
Supplement: Supplementary file 1 — Supplementary Material [file CLT2-12-e12139-s001.docx]

**Table 1** Clinical evaluation criteria of SAC

| Symptoms | Itch | 0 | None |
| --- | --- | --- | --- |
|  |  | 1 | Intermittent itching sensation |
|  |  | 2 | Continual awareness but without the desire to rub |
|  |  | 3 | Continual awareness with the desire to rub the eyes |
|  |  | 4 | Subject insists on rubbing eyes |
|  | Redness | 0 | None |
|  |  | 1 | Perhaps localized within some quadrant |
|  |  | 2 | More marked and diffuse reddening in the quadrants |
|  |  | 3 | Very marked and diffuse reddening in the quadrants |
|  | Tearing | 0 | None |
|  |  | 1 | Slightly humid eye |
|  |  | 2 | Some tears blow nose occasionally |
|  |  | 3 | Profuse tearing, tears rolling down cheeks |
|  | Foreign body | 0 | None |
|  | sensation | 1 | Occasionally feels sandy |
|  |  | 2 | Feels sandy daily |
|  |  | 3 | Occasionally look for foreign body |
|  | Burning sensation | 0 | None |
|  |  | 1 | Occasional |
|  |  | 2 | Daily with occasionally closing |
|  |  | 3 | Close eye daily |
| Ocular reactions | Palpebral conjunctiva | 0 | No manifestations |
|  | hyperemia | 1 | Dilatation of several vessels |
|  |  | 2 | Dilatation of many vessels |
|  |  | 3 | Impossible to distinguish  individual blood vessels |
|  | Palpebral conjunctiva | 0 | No manifestations |
|  | swelling | 1 | Localized edema |
|  |  | 2 | Diffuse mild edema |
|  |  | 3 | Diffuse marked edema |
|  | Bulbar conjunctiva | 0 | No manifestations |
|  | hyperemia | 1 | Dilation of several vessels |
|  |  | 2 | Dilation of many vessels |
|  |  | 3 | Vasodilatation of all vessels |
|  | Bulbar conjunctiva | 0 | No manifestations |
|  | chemosis | 1 | Partial conjunctival swelling |
|  |  | 2 | Diffuse thin chemosis |
|  |  | 3 | Cyst-like chemosis of entire  conjunctiva |
|  | Palpebral conjunctiva | 0 | No manifestations |
|  | follicle | 1 | 1-9 follicles |
|  |  | 2 | 10-19 follicles |
|  |  | 3 | 20 or more follicles |
|  | Palpebral conjunctiva | 0 | No manifestations |
|  | Papillae+ | 1 | Diameter 0.1-0.2 mm |
|  |  | 2 | Diameter 0.3-0.5 mm |
|  |  | 3 | Diameter ≥0.6 mm |

+ In cases having giant papillae, papillae and giant papillae should be graded simultaneously.
